# Trench MOS Schottky Diodes: A Physics-Based Analytical Model Approach to Charge Sharing

**DOI:** 10.3390/mi16010090

**Published:** 2025-01-14

**Authors:** Mohammed Tanvir Quddus, Alvaro D. Latorre-Rey, Zeinab Ramezani, Mihir Mudholkar

**Affiliations:** Power Solutions Group, Onsemi, Scottsdale, AZ 85250, USAmihir.mudholkar@onsemi.com (M.M.)

**Keywords:** charge sharing, trench MOS, Schottky diodes, analytical model

## Abstract

Trench MOS Barrier Schottky (TMBS) rectifiers offer superior static and dynamic electrical characteristics when compared with planar Schottky rectifiers for a given active die size. The unique structure of TMBS devices allows for efficient manipulation of the electric field, enabling higher doping concentrations in the drift region and thus achieving a lower forward voltage drop (VF) and reduced leakage current (IR) while maintaining high breakdown voltage (BV). While the use of trenches to push electric fields away from the mesa surface is a widely employed concept for vertical power devices, a significant gap exists in the analytical modeling of this effect, with most prior studies relying heavily on computationally intensive numerical simulations. This paper introduces a new physics-based analytical model to elucidate the behavior of electric field and potential in the mesa region of a TMBS rectifier in reverse bias. Our model leverages the concept of shared charge between the Schottky and MOS junctions, capturing how electric field distribution is altered in response to trench geometry and bias conditions. This shared charge approach not only simplifies the analysis of electric field distribution but also reveals key design parameters, such as trench depth, oxide thickness, and doping concentration, that influence device performance. This model employs the concept of shared charge between the vertical Schottky and MOS junction. Additionally, it provides a detailed view of the electric field suppression mechanism in the TMBS device, highlighting the significant effects of the inversion charge on the MOS interface. By comparing our analytical results with TCAD simulations, we demonstrate strong agreement, underscoring the model’s accuracy and its potential to serve as a more accessible alternative to resource-intensive simulations. This work contributes to a valuable tool for TMBS device design, offering insights into electric field management that support high-efficiency, high-voltage applications, including power supplies, automotive electronics, and renewable energy systems.

## 1. Introduction

Trench MOS Barrier Schottky (TMBS) diodes offer superior electrical characteristics compared to planar Schottky diodes for a given active die area [1,2,3,4,5]. TMBS diodes have gained significant attention for their ability to combine high efficiency with low power loss, making them ideal for high-performance applications in fields such as automotive electronics, power supplies, and renewable energy systems [4,6]. The incorporation of trenches in TMBS diodes allows for the reshaping of the electric field under reverse bias in the mesa region under the Schottky junction [7]. This reshaping facilitates higher doping in the drift region, resulting in a lower forward voltage drop (VF) and reduced leakage current (IR) while maintaining the same breakdown voltage (BV) as planar Schottky devices [8,9,10,11,12]. This electric field engineering also improves the diode’s thermal stability and enhances its switching characteristics, making TMBS diodes more reliable in high-temperature and high-frequency environments compared to planar Schottky diodes [13,14]. This advantage is significant for applications requiring high efficiency and reliability.

Despite the superior performance of TMBS diodes, the literature contains only a few analytical models that discuss the two-dimensional (2D) electric field distribution using Poisson’s equation solutions [15,16,17]. Existing models primarily focus on planar Schottky and trench-based devices without fully capturing the unique characteristics introduced by trench MOS structures, particularly in the complex electric field profiles within the mesa region. As such, many existing models lack the precision needed for effective TMBS design optimization, especially when high doping concentrations and trench structures are involved [18]. However, these models do not provide a comprehensive understanding of the electric field reshaping mechanism in the mesa region of trench devices. To address this gap, this paper aims to present a simple yet accurate analytical model that delves into the charge sharing concept and explains its impact on the reverse bias electric field shape, thereby elucidating the enhanced characteristics of TMBS devices.

**Figure 1:**
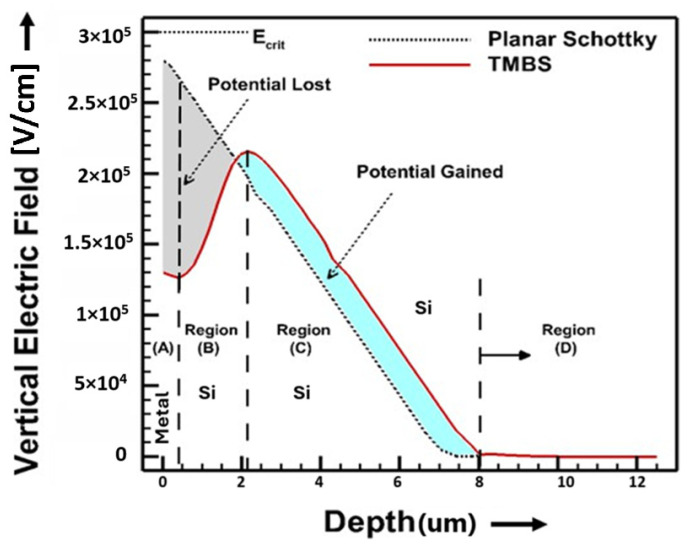
Vertical electric field in the middle of the mesa region of the TMBS device and a planar Schottky device.

The first section of this paper analytically derives the charge sharing mechanism, followed by the formulation of model equations that describe the different regions of the device. These equations capture the nuances of electric field distribution and doping concentration variations. Subsequently, we compare our analytical model with TCAD simulations to validate its accuracy. The comparison demonstrates a strong agreement between the model and TCAD data, confirming the reliability of our approach. This analytical validation is essential for ensuring that the proposed model can be used confidently in practical design scenarios, particularly in cases where simulation resources are limited or where rapid estimation is required [19]. Figure 1 shows the electric field along the center of the Mesa region in the vertical direction under reverse bias for a TMBS and planar Schottky rectifier. The electric field reshaping behavior is evident in the TMBS structure, where the electric field reaches a peak near the bottom of the trenches, reduces in value and finally increases near the surface. This behavior is analogous to an inversion of the drift region doping concentration from n-type to p-type in the mesa regions between the trenches, and finally shift back to n-type near the Schottky surface.

Understanding this behavior is crucial, as it confers several performance advantages to the TMBS structure over the planar structure. The unique electric field distribution in TMBS diodes enhances their performance in several ways. Firstly, the lower VF and fast switching translate to higher efficiency, which is essential for power-sensitive applications such as in power supplies and automotive electronics. Secondly, the reduced IR minimizes power loss, contributing to the overall energy efficiency of the device. Lastly, the ability to maintain a high BV while achieving these benefits makes TMBS diodes highly suitable for high-voltage applications. In summary, TMBS diodes represent an advanced solution in power semiconductor technology, providing a competitive edge in applications where both efficiency and reliability are paramount.

## 2. Charge Sharing Model

**Figure 2:**
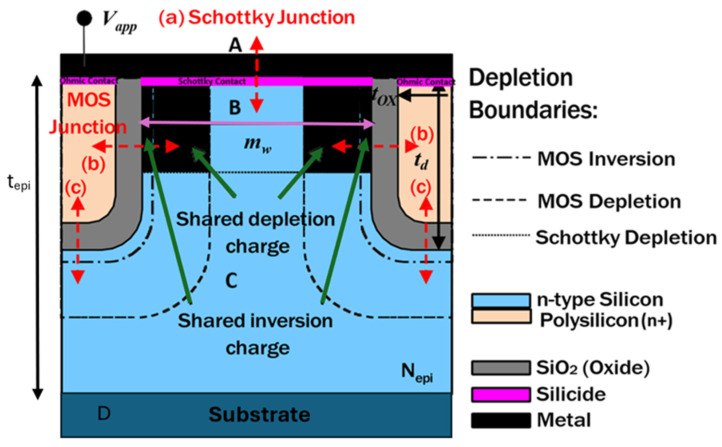
Active cell of a TMBS device with separated MOS inversion, MOS depletion and Schottky depletion region boundaries. m_w,_ t_d,_ and t_epi_ are mesa width, trench depth, and epi depth.

Figure 2 illustrates the active structure of a TMBS rectifier, which consists of three key junctions that support the electric field in the reverse direction. The first is a vertical junction between the Schottky barrier metal and the silicon mesa region (a). The second is a lateral junction formed between the MOS trench sidewall and the silicon mesa region (b). The third is another vertical junction located at the bottom of the trenches, connecting the MOS and the silicon drift region underneath it (c). These junctions work together to manage the electric field effectively, ensuring reliable device operation.

Each of these junctions plays a critical role in controlling the electric field, allowing for effective modulation of field intensity across the device. For the sake of simplicity, the rounding effect at the bottom of the trenches has been ignored in present analysis as it has a minor impact on the actual calculations, unless small radii are used.

Under reverse bias (V_app_ < 0) on the device, the depletion regions of the three junctions expand to support the applied voltage. The spatial distribution of these depletion regions is carefully engineered to prevent excessive electric field concentration at any single junction, which could otherwise lead to premature breakdown. In semiconductor devices, high electric fields at localized points can cause phenomena like avalanche multiplication or tunneling, both of which can result in irreversible damage to the device. To address this, engineers optimize the doping concentration of the semiconductor material to create a gradual and uniform transition between regions of differing electrical properties.

Moreover, the junction depths are precisely controlled to avoid abrupt interfaces that might cause sharp increases in the electric field. Geometric dimensions, such as the curvature of the junction edges or the thickness of insulating layers, are carefully designed to spread the electric field evenly. This uniform distribution reduces the likelihood of localized electric field “hotspots,” where the electric field intensity becomes disproportionately high.

By ensuring the depletion regions are spread out in a controlled manner, the device achieves a higher breakdown voltage, meaning it can operate reliably at higher voltages without failing. This engineering approach not only improves the robustness of the device but also enhances its efficiency, as the smoother electric field distribution reduces leakage currents and power losses. Ultimately, this precise control over depletion regions plays a crucial role in the performance, longevity, and safety of high-voltage electronic devices.

As seen in Figure 2, the depletion region of junctions (a) and (b) partially overlap, leading to shared depletion charge. This sharing effectively reduces the charge available for each junction, altering the overall electric field distribution. This shared charge distribution is fundamental to the device’s ability to support higher reverse voltages without compromising on breakdown voltage or increasing leakage current for a relatively higher doping concentration in the n-type Si mesa region.

In addition, the MOS capacitor operates in between full inversion and deep depletion, which not only forms a depletion region but also creates a thin inversion layer beneath the oxide. This thin inversion layer contributes to the overall electric field control within the device, providing an additional degree of modulation at higher applied biases. A part of the inversion charge is also shared with the Schottky junction, resulting in a reduction in inversion charge available for the MOS capacitor. The sharing of the inversion charge between the MOS capacitor and the Schottky junction also increases the net depletion width of the MOS capacitor, as the capacitor deviates from full inversion to a partial inversion mode. This shift impacts the overall electric field distribution and performance of the device, as the MOS capacitor now operates with an expanded depletion region and reduced inversion charge. This shift in operation results in a dynamic adjustment of the electric field across the MOS structure, optimizing the device for higher efficiency by effectively managing the electric field intensity. This expanded depletion region and reduced inversion charge in the MOS capacitor impact the overall electric field distribution and performance of the device, as the MOS capacitor now operates with an expanded depletion region and reduced inversion charge, enabling better control over leakage and breakdown characteristics under high reverse bias conditions.

In order to develop a model for the shared charge between the MOS trench capacitor and the Schottky junction, the charge components for each distinct region within the device structure are first calculated in isolation. This segmented approach allows for a focused analysis of individual contributions to the electric field and potential within each region, making it easier to understand the effects of various device design parameters, such as trench depth and oxide thickness. Once these charges are calculated separately, the principle of superposition is applied to combine the effects of each region, resulting in a comprehensive model that reflects the behavior of the entire TMBS structure. Superposition, as used here, provides a straightforward way to account for complex interactions between the depletion regions of the Schottky and MOS structures without resorting to computationally intensive simulations. This analysis of using superimposition to combine the effects of shared depletion region has been presented in literature for RESURF devices [20,21,22,23]. In RESURF structures, similar principles of charge sharing and field modulation are used to control electric field distribution and enhance breakdown voltage, making the methodology applicable to TMBS design as well. The inversion charge, $Q_{inv}$, of a bulk MOS capacitor is given by [24]:

(1) $$Q_{inv}=2\;v_{th}C_{OX}.L\left(\frac{\sqrt{2k_{s}\varepsilon_{0}n_{i}}}{2v_{th}C_{OX}}.\exp\{-\frac{V_{app}-V_{fb}}{2v_{th}}\}\right)$$

where $v_{th}$ is the thermal voltage, $C_{OX}$ is the oxide capacitance per unit area, L (.) is the Lambert function [25], $V_{app}$ is the applied reverse voltage and $V_{fb}$ is the flat-band voltage of the MOS capacitor. Constants $k_{s},\varepsilon_{0},\;and\;n_{i}$ refer to relative permittivity factor of silicon, permittivity of vacuum and intrinsic carrier concentration of silicon, respectively.

In the TMBS structure, the inversion charge near the MOS capacitor oxide plays a pivotal role in determining the overall electric field distribution within the device. This inversion charge can be treated as a volume charge, characterized by a concentration of $Q_{inv}$/$L_{D}$ where $Q_{inv}$ is the surface concentration and $L_{D}$ is the Debye length [26]. This volumetric treatment of inversion charge is crucial for accurately modeling the field behavior near the MOS capacitor, as it allows for a more refined depiction of the charge dynamics close to the oxide interface.

The inversion charge near the Schottky junction contributes towards the total charge available for the depletion width of the Schottky junction near the SiO_2_/Si interface, effectively altering the electric field and potential in this region. By accounting for the inversion charge as a distributed volume charge, this model can capture the interaction between the MOS and Schottky junctions more accurately. This interaction is essential in understanding how trench structures in TMBS devices help suppress leakage currents and improve breakdown voltage, ultimately enhancing the performance of the device. The inversion charge can thus be modeled as:

(2) $$W_{schinv}=\sqrt{\frac{{2k_{s}\varepsilon_{0}(V}_{bi}-V_{app})}{q(N_{epi}+\frac{Q_{inv}}{L_{D}})}}$$

where N_epi_ is the doping concentration of the drift region. Charge used in this region is:

(3) $$\Delta Q_{inv}=Q_{inv}.\eta_{inv}=Q_{inv}.{2W}_{schinv}/t_{d}$$

Therefore, the effective inversion charge of the MOS capacitor is given by:

(4) $$Q_{inveff}=Q_{inv}-\Delta Q_{inv}=Q_{inv}(1-\eta_{inv})$$

The effective inversion charge, Q_inveff_, represents the inversion charge of the MOS capacitor after accounting for the portion of the charge (ΔQ_inv_) utilized in the depletion region near the Schottky junction. This reduction occurs because some of the inversion charges near the oxide interface contributes to extending the depletion width of the Schottky junction (W_schinv_), which affects the electric field distribution. By considering this reduction, the model provides a clearer understanding of the interplay between the MOS trench capacitor and the Schottky junction, as well as the effects on the device’s electric field modulation and performance.

It is emphasized here that this model provides a first-order approximation of the charge-sharing mechanism, with the charge values averaged over the entire charge distribution. This approximation simplifies the complex interactions between the MOS trench capacitor and the Schottky junction, offering a practical way to capture the essential behavior of the device without the need for highly detailed, depth-dependent calculations. By focusing on an averaged charge distribution, this approach helps streamline the analysis, making it more accessible for initial design and optimization.

However, it is important to note that this model does not represent the actual depth-dependent distribution of charges or potential within the TMBS structure. Instead, it serves as a foundational model aimed at elucidating the primary charge-sharing mechanism at work, rather than capturing the intricate variations in charge or potential across different depths. This first-order approach provides a balance between analytical simplicity and accuracy, delivering insights into the core behavior of the TMBS device while leaving room for more detailed modeling in future studies if necessary.

**Figure 3:**
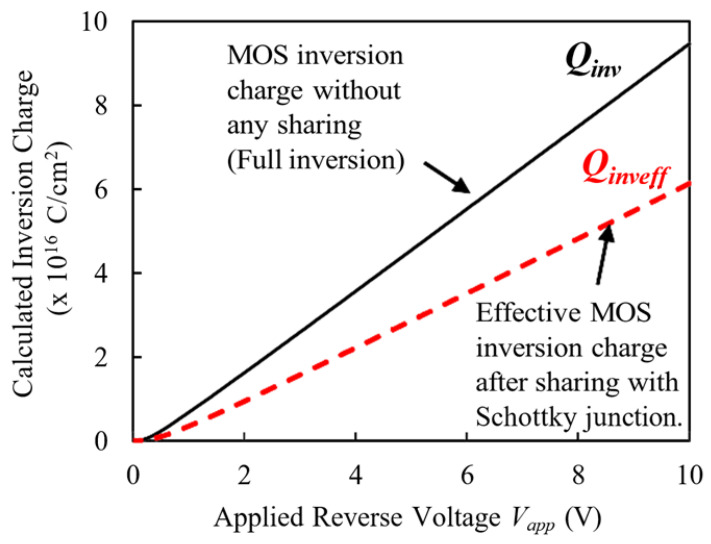
Calculated inversion charge for a MOS capacitor and reduction in inversion charges due to charge sharing with vertical Schottky junction.

The reduction in effective inversion charge is evident from Equation (4) and is shown in Figure 3.

This reduction plays a critical role in modifying the overall charge dynamics within the TMBS structure, directly influencing the behavior of the MOS capacitor’s depletion region. As the effective inversion charge decreases, the depletion region of the MOS capacitor is allowed to expand with increasing reverse bias. This is a notable deviation from the behavior observed in conventional bulk MOS capacitors, where the depletion region stabilizes once strong inversion is reached, effectively limiting further expansion.

In contrast, the shared inversion charge in the TMBS structure results in a broader depletion region that continues to grow as the reverse bias increases. This continuous expansion of the depletion region is significant, as it alters the electric field distribution within the MOS capacitor, enhancing the device’s ability to manage higher reverse voltages effectively. Additionally, this behavior affects the interaction between the MOS capacitor’s depletion region and that of the Schottky junction, particularly in the overlapping region shown in Figure 2. In this region, the depletion regions of both the MOS capacitor and the Schottky junction partially merge, creating a complex, shared depletion profile that contributes to improved electric field reshaping. This overlapping depletion also helps to reduce the peak electric field near the Schottky junction, enhancing breakdown characteristics and lowering leakage current under high reverse bias conditions. The bias dependent depletion width of the MOS capacitor due to reduction in $Q_{inv}$ can be written as:

(5) $$W_{MOSeff}=\frac{{k_{s}t}_{OX}}{k_{OX}}[\sqrt{1+\frac{{2(V}_{app}+Q_{inveff}/C_{OX})}{V_{o}}}-1]$$

where *K_ox_* is the relative permittivity factor of SiO_2_, *t_ox_* is the oxide thickness as indicated in Figure 2 and *V*_0_ is the body factor given by [26]:

(6) $$V_{o}=-\frac{{qk_{s}\varepsilon_{0}N}_{epi}}{{C_{OX}}^{2}}$$

**Figure 4:**
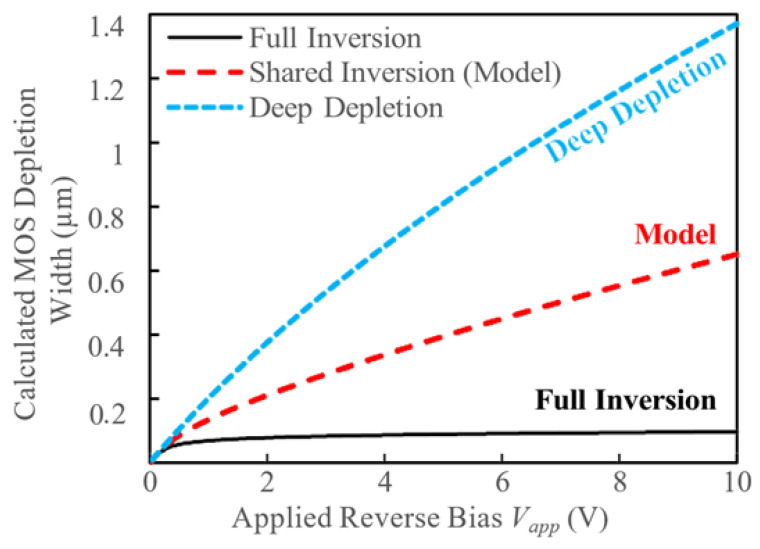
Calculated depletion width for the MOS capacitor in full inversion, weak inversion according to Equation (5) and deep depletion.

The variation in the MOS depletion region with applied reverse bias V_app_ for the case of full inversion ($Q_{inv}$), effective inversion ($Q_{inveff}$) and deep depletion ($Q_{inv}$ = 0) is shown in Figure 4. This variation highlights the MOS capacitor’s adaptive depletion behavior under different inversion conditions, illustrating how its depletion width shifts dynamically in response to changes in the inversion charge state.

The width of the depletion component of the MOS capacitor falls between the full inversion and deep depletion regimes, striking a balance that results in a moderate depletion width, tailored to the TMBS structure’s field requirements. This intermediate depletion width modifies the charge available for the Schottky depletion region, affecting the overall field distribution and reverse bias characteristics. The interplay between the MOS and Schottky depletion regions is crucial for optimizing the electric field distribution in the mesa region, as it allows the device to manage higher reverse biases while maintaining a lower leakage current and improved breakdown performance. Under reverse bias, the charge provided by the Schottky depletion region to support the applied bias in the mesa region away from the interface is given by:

(7) $$Q_{sch\;}={qN}_{epi}W_{sch}$$

where the Schottky depletion width W_sch_ is given as:

(8) $$W_{sch}=\sqrt{\frac{{2k_{s}\varepsilon_{0}(V}_{bi}-V_{app})}{{qN}_{epi}}}$$

However, due to the MOS capacitor depletion region overlap, the available charge for the Schottky junction is reduced by a factor $\Delta Q_{sch}$ given by:

(9) $$\Delta Q_{sch}=\frac{{qN}_{epi\;}.2W_{MOSeff}\;\;}{m_{w}}=Q_{sch}.\eta_{sch}$$

Thus, the effective charge available for the Schottky depletion region in the mesa is given by:

(10) $$Q_{scheff\;}={Q_{sch\;}-\Delta Q_{sch}=Q}_{sch}(1-\eta_{sch\;})$$

where η_sch_ is a measure of the degree of shared Schottky depletion charge in the mesa region and WMOSeff is effective width of the MOS region.

It is evident from Equation (9) that as the applied reverse bias V_app_ is increased, η_sch_ will increase due to an increase in the MOS capacitor depletion region width. This relationship indicates that as the reverse bias rises, the MOS depletion width dynamically adjusts, impacting the charge sharing and electric field distribution within the TMBS structure. At a sufficiently high V_app_, effective charge available for the Schottky junction in between the trench’s changes sign according to Equation (10) and behaves like a P-type material instead of N-type. This inversion of charge polarity within the drift region near the Schottky junction has significant implications for the device’s electric field behavior, as it effectively reverses the charge dynamics that govern field distribution. This inverting of the drift region charge for the Schottky junction causes the reduction in vertical electric field in between the trenches. This phenomenon is qualitatively described as the “pinch-off” effect, where the Schottky mesa electric field is increasingly confined by the trench regions, reducing its intensity and spread across the mesa. The developed equations provide a first-order quantitative model for this pinch-off effect, offering a simplified yet effective means to capture the impact of charge inversion and trench confinement on the electric field distribution. This model thus enables a more controlled manipulation of electric field intensity, which can be advantageous in optimizing device performance under high reverse bias conditions, enhancing both breakdown resilience and leakage reduction.

## 3. Model Results and Discussion

**Figure 5:**
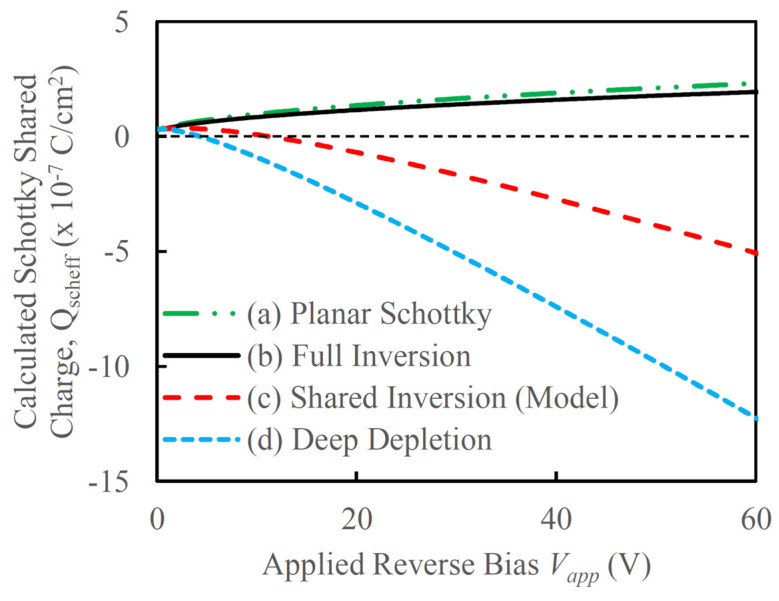
Calculated charge available for the Schottky depletion region in the mesa region between trenches for (**a**) planar Schottky, (**b**) TMBS with MOS capacitor assumed under full inversion, (**c**) TMBS with shared inversion and (**d**) TMBS with MOS capacitor assumed under deep depletion (no inversion).

Figure 5 shows the charge in the mesa region between the MOS trenches available for the Schottky depletion region for four different scenarios: (a) planar Schottky junction without any charge sharing, (b) Schottky junction with the MOS capacitor in full inversion, (c) Schottky junction with MOS capacitor with shared inversion charge as proposed in the present work according to Equations (4) and (10), and (d) Schottky junction with MOS capacitor with deep depletion (Q_inv_ = 0). Each scenario highlights different modes of interaction between the MOS and Schottky junctions, offering a comparative view of how varying inversion charge conditions influence the charge distribution within the Mesa region. For case (a), it is easy to understand that the total depletion charge available for the Schottky junction increases proportionally to $\sqrt{V_{app}}$ as all the depletion charge is due to the Schottky junction. Since there is no charge sharing in this planar configuration, the depletion region’s behavior aligns with that of a traditional Schottky diode, where the depletion charge is directly related to the applied reverse bias, resulting in a simple square root dependency.

With the presence of the vertical MOS trenches, the charge in the depletion region under the Schottky junction is shared between the two junctions. This shared charge mechanism plays a critical role in determining the overall electric field profile and charge distribution within the TMBS structure, as it allows for a dynamic adjustment based on the state of the MOS capacitor. Assuming full inversion of the MOS capacitor, the width of the MOS depletion region becomes pinned once the capacitor reaches inversion. This behavior occurs because any additional applied bias primarily serves to increase the inversion charge at the surface, rather than expanding the depletion width. This effect can be observed in Figure 5, case (b), where a slight reduction in the total charge available for the Schottky junction is seen when a full inversion charge is assumed for the MOS capacitor. This configuration leads to a relatively stable electric field in the MOS region, as the inversion charge compensates for increased bias, limiting the impact on the Schottky depletion region. On the other hand, if the MOS capacitor is assumed to be in full depletion, then the width of the MOS depletion region continues to increase with V_app_, significantly decreasing the charge available for the Schottky junction. In this scenario, the depletion region’s expansion reduces the overall inversion charge, which in turn affects the electric field intensity and distribution under the Schottky junction, allowing for a different mode of charge control. However, in practical operation, the MOS capacitor is typically in an inversion state, with a portion of the inversion charge being shared with the Schottky junction as described by Equation (2). This partial sharing of inversion charge creates a hybrid condition where the MOS capacitor operates with a moderated depletion width and inversion charge. This configuration allows for a balance between field suppression and charge distribution across both junctions, enhancing the device’s ability to handle higher reverse biases efficiently while maintaining control over leakage and breakdown characteristics.

Once the effective charge utilized by the Schottky depletion region between the trench MOS capacitors has been calculated using Equation (10), the electric field profile along the center of the mesa region can be determined with relative ease. To simplify this calculation, a constant effective charge distribution is assumed across various regions of the device, enabling a streamlined approach to modeling the electric field without requiring complex depth-dependent calculations.

The vertical electric field along the center of the mesa region can be divided into four distinct regions: Region (A), located near the Schottky surface within the zero-bias depletion width, represents the initial field near the surface interface where the Schottky barrier predominantly influences the field distribution; Region (B), which extends from the zero-bias depletion width down to the bottom of the trenches, serves as a critical area where the charge sharing between the MOS capacitor and Schottky junction takes place; Region (C), from the bottom of the trenches to the bottom of the drift region, represents the portion of the field influenced by the underlying drift region characteristics; and a fourth region, Region (D), which is only present if the device enters punch-through mode in the highly doped substrate (see Figure 1).

In Region B, the majority of the charge-sharing effect occurs, as this is the area where the trenches impose significant field suppression. The charge redistribution within this region plays a vital role in modulating the electric field intensity, thereby preventing excessive field concentration near the Schottky junction and enhancing breakdown performance. Region B, therefore, is the primary area of electric field suppression, as the interaction between the MOS trench capacitors and the Schottky depletion region reduces the net electric field, allowing the TMBS structure to handle higher reverse biases with improved efficiency and stability.

In order to use the shared charge model to estimate the vertical electric field profile along the center of the mesa region, effective charges for each segment, Regions (A) through (D), are first calculated. These calculations are based on the equations developed in Section II, which provide a structured approach to analyzing the charge distribution across different regions of the TMBS structure.

In Region (A), located near the Schottky junction interface, most of the charges are assumed to be concentrated within the Schottky junction itself. This initial region is particularly important, as it sets the foundation for the electric field distribution across the mesa, influencing how the field interacts with adjacent regions. The boundary of Region (A) is approximated to the zero-bias depletion width of the Schottky junction, denoted as (W_sch_(V_app_ = 0)), which represents the depletion width under zero applied bias. This assumption simplifies the modeling by providing a well-defined boundary condition, ensuring that the field calculations in Region (A) accurately capture the initial electric field near the Schottky surface. By establishing this boundary at the zero-bias depletion width, we ensure that Region (A) effectively encompasses the primary contribution from the Schottky junction, allowing for a straightforward calculation of the initial electric field. This setup also facilitates the integration of charge contributions from subsequent regions, creating a coherent electric field profile across the mesa that reflects the cumulative effect of charge sharing between the MOS trench capacitors and the Schottky junction.

At low biases, the MOS capacitor depletion region extends partially into the mesa region, and the formulation for Q_scheff_ according to Equation (10) is valid. In this regime, the MOS depletion region remains sufficiently narrow, allowing each MOS capacitor to operate independently, with limited interaction between adjacent depletion regions. However, at higher applied biases, the MOS depletion region expands further, eventually exceeding half of the mesa width. At this point, the depletion region from one MOS capacitor begins to overlap and coupled with the depletion region from the adjacent MOS capacitor on the other side of the mesa. This effect is similar to a double MOS-gated structure, where the two depletion regions work in tandem, influencing the electric field distribution and charge sharing within the mesa region. This coupling introduces additional complexity in the field profile, as the shared electric field from both MOS capacitors now interacts more significantly with the Schottky depletion region, altering the overall charge distribution and field characteristics.

To accurately account for this coupling effect of the two overlapping MOS depletion regions, along with the Schottky depletion region, a coupling factor is defined as follows:

(11) $$\alpha_{coupling}=\frac{min\;[m_{w},\;2W_{MOSeff}]\;}{2W_{MOSeff}}$$

where $\alpha_{coupling}$ varies between 0 and 0.5 for low to high V_app_. Using the coupling factor, the charge density for each region can be calculated as

(12) $$\rho_{A}={{qN}_{epi\;}.\;\alpha }_{coupling}$$

(13) $$\rho_{B}={{qN}_{epi\;}.\;\alpha }_{coupling}(1-\eta_{sch})$$

(14) $${\rho_{C}=qN}_{epi\;};\;{\rho_{D}=qN}_{sub}$$

Using the developed effective charge equations according to (12)–(14), the electric field can be calculated by integrating the assumed constant charge distributions to provide a first order electric field behavior. This approach simplifies the calculation by treating the charge distribution as uniform within each region, allowing for a straightforward integration that yields a clear profile of the electric field’s behavior along the device depth. The various electric field as functions of depth x can be calculated as [26,27]:

(15) $$E_{x}=\int_{x_{1}}^{x_{2}}\rho \;dx$$

where *x*_1_ and *x*_2_ are the surface and the vertical length of the device. This formulation allows for efficient estimation of the electric field within the TMBS structure, bypassing the complexity of non-uniform charge distributions while still capturing the essential field dynamics.

**Figure 6:**
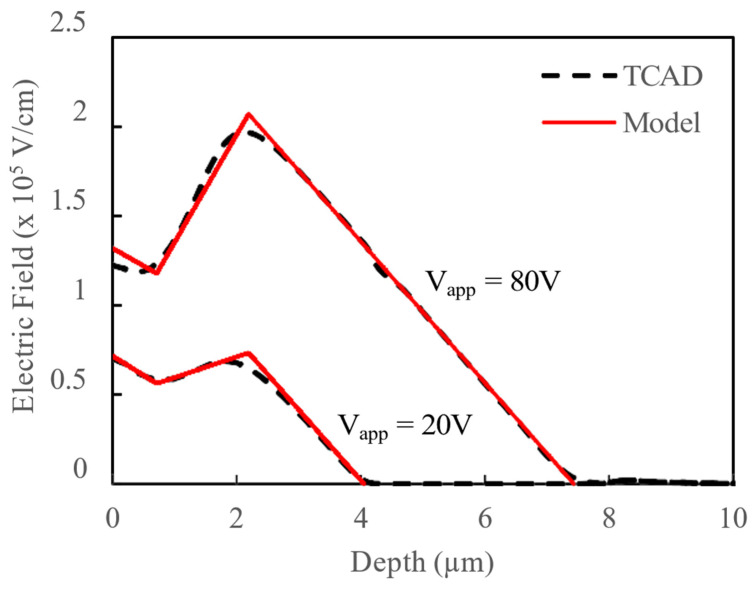
Comparison of electric field obtained from TCAD simulation and proposed analytical model for applied reverse bias of 20 V and 80 V.

The breakdown voltage of the device is determined by the integral of the electric field along its depth. Optimizing design parameters, such as trench depth, trench width, mesa width, doping concentration, oxide thickness, and the thickness of the n-type Si region, can reduce the peak electric field in the n-type Si mesa region, as illustrated in Figure 6. This reduction in peak electric field not only increases BV by minimizing leakage through the suppression of the image force barrier lowering effect in the Schottky barrier, but also allows for higher doping concentration in the n-type Si region at a given BV, significantly enhancing the device’s electrical performance in forward conduction and switching applications.

Figure 6 shows the comparison between the simulated vertical electric fields using TCAD with the electric field distributions calculated using the proposed analytical model for reverse bias V_app_ values of 20 V and 80 V. The comparison shows that the analytical model closely aligns with the TCAD simulations across all regions of interest, demonstrating its effectiveness in capturing the electric field profile under both low and high reverse bias conditions. While the model provides a straightforward approximation of the electric field, some minor discrepancies between the calculated electric field and the 2D TCAD simulations are observed. These differences underscore the utility of the model in understanding the electric field reshaping behavior enabled by the trench structures, as it captures the general trend and effects of the trenches on field distribution while simplifying computational effort. This balance of simplicity and accuracy makes the analytical model an efficient alternative to full-scale simulations for initial design evaluations and field optimization.

In Table 1 key parameters used in the model are mentioned [22].

## 4. Conclusions

A simple analytical model has been introduced in this paper, focusing on the shared charges within the mesa region of TMBS devices, to elucidate the mechanism of electric field suppression in trench-based structures. This model provides a structured approach to understanding how the electric field is modulated within trench structures, addressing a key aspect of TMBS and other trench-based devices that is often challenging to capture accurately with complex simulation tools alone. The model is versatile and can be applied to various trench-based devices, such as MOSFETs and IGBTs, making it a valuable tool for device engineers and researchers working on a broad spectrum of power semiconductor technologies. By leveraging average shared charges, the paper provides insights into how charges are manipulated within the mesa region and how the electric field varies with different device parameters, such as trench depth, doping concentration, and oxide thickness. This flexibility enables the model to serve as a predictive tool, offering guidance for design optimization in a variety of trench-based structures. Despite the simplifications and assumptions inherent in the model formulation, the study demonstrates that the model accurately predicts the electric field in the mesa region of TMBS devices. This accuracy reinforces the model’s applicability in real-world scenarios, where quick approximations are often needed during the initial stages of device design. The accuracy of the model has been validated through comparisons with TCAD 2D simulations of identical device structures, highlighting the model’s effectiveness in capturing the essential physics of trench-based devices. Such comparisons confirm that the model is not only theoretically sound but also practically reliable for predicting field behavior under different operational conditions. Moreover, this analytical approach offers a clear understanding of the electric field reshaping behavior enabled by trenches, which is crucial for optimizing device performance in various applications, including high-efficiency power supplies, automotive electronics, and renewable energy systems. By providing a straightforward yet effective method to analyze electric field suppression, this model significantly contributes to the broader understanding and advancement of trench-based semiconductor devices. It serves as a foundational tool for both academic research and practical device engineering, facilitating innovations that rely on precise field control and charge management in trench-based architecture.

## Figures and Tables

**Table 1:** Key parameters for TMBS analytical model.

Parameter	Symbol	Value	Unit
Relative permittivity of silicon	k_s_	11.89	-
Relative permittivity of oxide	k_ox_	3.9	-
Oxide thickness	t_ox_	0.38 × 10^−4^	cm
Permittivity of vacuum	ε_0_	8.854 × 10^−14^	F/cm
Silicon doping concentration	N_epi_	2.6 × 10^15^	cm^−3^
Electronic charge	q	1.602 × 10^−19^	C
Work function of Schottky metal	Φ_M_	4.75	eV
Electron affinity of silicon	χs	4.05	eV
Boltzmann constant	k	8.617 × 10^−5^	eV/K
Intrinsic carrier concentration	n_i_	1.5 × 10^10^	cm^−3^
Temperature	T	300	K
Schottky trench depth	t_d_	2.0	um
Epi depth	t_epi_	8.0	um
Epi Ref depth	t_epiref_	10	um
Mesa Width	m_w_	1.4 × 10^−4^	cm

## Data Availability

Data are included in this paper.
